# Indicators of deterioration in young adults with serious mental illness: a systematic review protocol

**DOI:** 10.1186/s13643-018-0781-y

**Published:** 2018-08-16

**Authors:** Lindsay H. Dewa, Elizabeth Cecil, Lynne Eastwood, Ara Darzi, Paul Aylin

**Affiliations:** 1 NIHR Imperial Patient Safety Translational Research Centre, Imperial College London, London, UK; 2 School of Public Health, Imperial College London, Reynolds Building, St Dunstan’s Road, London, W6 8RP UK; 3 West London Mental Health Trust, London, UK

**Keywords:** Serious mental illness, Mental health, Young adults, Deterioration, Indicators

## Abstract

**Background:**

The first signs of serious mental illnesses (SMIs) including schizophrenia, bipolar disorder and major depression are likely to occur before the age of 25. The combination of high prevalence of severe mental health symptoms, inability to recognise mental health deterioration and increased likelihood of comorbidity in a complex transitional young group makes detecting deterioration paramount. Whilst studies have examined physical and mental health deterioration in adults, no systematic review has examined the indicators of mental and physical deterioration in young adults with SMI. The study aim is to systematically review the existing evidence from observational studies that examine the indicators of mental and physical deterioration in young adults with SMI and highlight gaps in knowledge to inform future research.

**Methods:**

Seven databases including CINHAL, MEDLINE, Embase, PsycINFO, Health Management Information Consortium, Cochrane databases and Web of Science will be searched against five main facets (age, serious mental illness, sign, deterioration and patient) and a subsequent comprehensive list of search terms. Searches will be run individually in each database to reflect each unique set of relevant subject headings and appropriate MeSH terms. Inclusion and exclusion criteria were developed and refined by the research team. Two reviewers will participate in each search stage including abstract/title and full text screening, data extraction and appraisal, to ensure reliability. A narrative synthesis of the data will also be conducted.

**Discussion:**

This systematic review will likely make a significant contribution to the field of mental health and help inform future research pertaining to interventions that help highlight deteriorating patients. This may vary depending on the patient group, mental illness or deterioration type.

**Systematic review registration:**

PROSPERO CRD42017075755

**Electronic supplementary material:**

The online version of this article (10.1186/s13643-018-0781-y) contains supplementary material, which is available to authorized users.

## Background

Serious mental illness (SMI) include conditions that are usually debilitating to the brain, behaviour and day to day functioning. Examples of SMI include major depression, bipolar disorder and psychotic disorders [1]. Whilst 5.8% of the general population has SMI at any one time, three quarters of serious mental health problems develop before the age of 25, with young adults at particular risk of developing first-episode psychosis [2, 3]. Prevalence of psychotic disorders and bipolar disorder in young adults (16–24 years of age) over 12 months is 0.5 and 3.4% respectively [4]. The symptoms of SMI can emerge during their transition into adulthood which can be a particularly difficult time because of various life changes that can occur. For instance, going to university in a different city, starting new employment or starting new serious relationships [5] are likely to cause stress and can be even more challenging if experiencing SMI. Consequently, detecting deteriorating mental health is particularly difficult for this young population.

Deterioration of health is the process in which our mental and physical health becomes progressively worse over time. Research has identified indicators of mental health deterioration or re-/hospitalisation across several SMIs including schizophrenia [6, 7], bipolar disorder [8, 9] and major depressive disorder [10]. Severe symptoms of deterioration can include mania, psychosis, aggressive behaviour, suicidal thoughts and behaviour and are well-known risk factors for inpatient admission [7, 11]. Indeed, over two thirds of people who died by suicide between 2004 and 2014 were under the care of mental health services before their death [12]. Patients with SMI are also at increased risk of asphyxiation during restraint, rapid tranquilization, developing obesity and heart conditions [13]. Moreover, other health issues including pain, severe insomnia, CVD, gastrointestinal disease, respiratory disorders and substance misuse disorders are also common in this patient group [14]. Despite these well-known associative factors, physical health deterioration in people with SMI is often missed. Comorbidity is common in young adults [14, 15], making the detection of mental health deterioration difficult. It is therefore important to intervene early in this transition period to avoid the worsening of symptoms, possible hospitalisation and potential unsafe behaviours. Indeed, early identification of deterioration and rapid response can significantly reduce adverse events, including, in some cases, suicide [16].

Early strategies that help recognise onset and deterioration of SMI are extensive (e.g. [17, 18]); they range from self-report subjective assessments, to family and friends noticing changes in mood and behaviour, to face-to face general practitioner (GP) assessments to, technology-based assessments methods. GPs are in a good position to intervene early in young people but the majority of mental disorders go undetected [19]. This may be because of stigma in raising a mental health issue, or what the GP will say about them. Therefore, it is not surprising that young people do not feel that they have autonomy and control over their own mental health. Young people may also not be aware of when their mental health starts to deteriorate and early warning signs for relapse. It is possible that the high prevalence of mental health symptoms [2, 3], inability to recognise mental health deterioration and unwillingness to disclose impromptu concerns to healthcare professionals indicates the need for routine monitoring of mental health [19].

Detecting deterioration in mental health is therefore vital to providing better care for patients with SMI in the community and in healthcare settings. To our knowledge, only one publication has reviewed research on the deterioration of both mental and physical health in adults with mental ill-health [16]. It provided an overview of the current knowledge base, gaps that could be addressed by the Australian Commission and whether national criteria on physical deterioration could be applied to mental health. Factors relating to a deterioration in this population included self-harm, suicidality, anxiety and aggression [16]. Whilst the review was extensive, it was not specifically on young adults nor were young adults identifiable from each included study; it only focused on current inpatients in acute settings and it had methodological limitations. Methodological issues included a lack of adherence to PRISMA guidelines, quality assessment of studies and search strategy detail. Considerations are therefore multifaceted. Whilst research has investigated the physical and mental health deterioration in adults, no systematic review has examined the indicators of mental and physical deterioration in young adults with SMI. Consequently, there is a need to conduct a high-quality systematic review that exemplifies robust methodology in this area and covers young adult patients. The defined age range for young adults varies from study to study. We have chosen to focus on 18–25 year olds as this is the typical “transition” period from adolescence to younger adults. This period is recognised by general health, mental health and government strategies as crucial for targeting appropriate care and help [20, 21], namely detecting the possible worsening of mental health. The study aim is to systematically review existing evidence from observational studies that examine the detection of indicators of mental and physical deterioration in young adults with SMI over time and to highlight gaps in knowledge to inform future research. Our objectives are therefore to (1) describe indicators that detect deterioration in the cohort over time, (2) evaluate the quality of these studies and (3) assess the evidence associated with these indicators to detect deterioration.

## Methods

This is a systematic review of studies describing indicators of deterioration in young adults with SMI through observational studies. This protocol is written up according to the PRISMA guidelines (Additional file 1).

### Definitions

We will identify any indicators of deterioration associated with primary outcomes. Deterioration refers to “a condition that is gradually worsening” [22]. In this review, an indicator of mental health deterioration will be defined as a “change for the worse to mental state”, measured by negative changes in mood, behaviour, affect, thought, perception and cognition [16]. Physical deterioration will be defined as a change in clinical state to worse clinical state, which increases individual risk of morbidity. Therefore, the primary outcomes for mental health deterioration is deaths by suicide, attempted suicide, other self-harm, aggression and other harms to others, the use of restraint (including repeated and high-dose PRN) and seclusion and premature discharge from acute facilities [16]. Physical deterioration outcomes will include all-cause mortality, organ dysfunction, protracted hospital stay, disability and worsening of symptoms on a reliable scale [23].

### Search strategy and selection criteria

Cumulative Index to Nursing and Allied Health Literature (CINAHL), MEDLINE, Embase, PsycINFO, Health Management Information Consortium (HMIC), Cochrane databases and Web of Science databases will be searched from 1991 to October 2017 against a comprehensive list of search terms (Table 1). A search strategy will be developed over time in collaboration with the research team (PA, EC, LH) and two resident librarians (Additional file 2). Searches will be run individually in each database to reflect each unique set of relevant subject headings and appropriate MeSH terms. Grey literature will be sought through OpenGrey, the first 10 pages of Google and Google Scholar. For comprehensiveness, the reference lists of key publications and any key review will also be searched.

**Table 1:** Search terms against each key facet

Age	Serious mental illness	Sign	Deterioration	Patient
Exp young adult/	Exp bipolar disorder/	Indicat*	Declin*	exp patient/
Exp adolescent/	bipolar disorder*	Warn*	Deteriorat*	patient*
young adult*	Exp schizophrenia/	MEWS	Worse*	Inpatient*
Adolescen*	schizophrenia	Predict*	Downfall	in-patient*
young person*	Exp psychotic disorder/	Detect*	Weak*^`^	outpatient*
young people	psychotic disorder*	Sign*	Wane*	out-patient*
youth*	Exp major depressive disorder/	Measure*	Descen*	
Exp young adult/	major depression	Gauge	Laps*	
	major depressive disorder*	Index	Dip*	
	exp depressive disorder/	Criteria*		
	exp depression/	Highlight*		
	Schizoaffective*	Monitor*		
	Psychosis*	Symptom*		
	mania	Signal*		
	Serious mental illness*	Diagnos*		
		Characteristic*		
		Alarm*		
		Alert*		
		Caution*		
		Forewarn*		
		Trigger*		
		Risk*		
		Factor*		

*truncation

Ten percent of title and abstracts and 100% of full-text publications will be screened against selection criteria described below by two independent reviewers. Any disagreement will be resolved through discussion between two reviewers and a third reviewer (PA) until consensus is achieved. Inter-rater agreement will be calculated using the kappa statistic in Statistical Package for Social Sciences (SPSS Statistics 25); the agreement level must be substantial (*k* = 0.60–0.80).

Search terms were developed over a series of iterative rounds with the research team (PA, EC, LH) and two institutional librarians. Search terms will cover five main facets: serious mental illness, age, sign, deterioration and patient (see Table 1). Inclusion and exclusion criteria will be organised using the PICOS (population, intervention, comparator, outcomes, study design) strategy and include:

- ▪Population—young people (18–25 years of age) including patients, inpatients and outpatients
- ▪Population—young people (18–25 years of age) with serious/severe mental illness, specific disorders will include major depressive disorder, bipolar disorder and schizophrenia and related psychotic disorders [1]. In addition, study author description states “severe/serious mental illness”.
- ▪Intervention—no restriction
- ▪Comparator—no restriction
- ▪Outcome— signs of mental and physical health deterioration. “Signs” of mental deterioration are changes in mood, behaviour, affect, thought (stream, form and content), perception and cognition (mentor and orientation). Physical health can include health status, physical fitness, physical health and quality of life. Deterioration of physical health can be putting on weight, pain and poor health-related quality of life [14].
- ▪Study design—observational studies that include case-control, cohort or cross-sectional studies.
- ▪Setting—no restriction
- ▪Timing—1991 to present day

### Further inclusion criteria

- ▪Population—articles that mention young adults and the age group is defined as outside the age parameter (18–25) but individual ages can be identified separately. For example, publications covering the 16–24 age group collectively will be excluded but publications that isolate age within the given population (e.g. 18, 19, 20, 21, 22, 23 and 24) will be included.
- ▪Outcome—physical health deterioration will only be included if it is found in publications covering young adults with SMI
- ▪Outcome—study aim must centre on detecting signs of deterioration in young adults with SMI

### Exclusion criteria

- ▪Limit—non-English language
- ▪Population—articles that mention young adults but the age group is defined as outside the age parameter. For example, 16–24 years old as an overarching age group will be excluded.
- ▪Study design—conference abstracts, book chapters, protocols
- ▪Study design—editorials/opinion/discussion pieces/commentaries/clinical case reviews/reviews
- ▪Outcome—study aim centres on detecting signs of physical health deterioration in young adults but who do not have a SMI

### Data extraction

A standardised data extraction method will be used. Author, year of publication, country, design (main: quantitative/qualitative and specific), type/number/characteristics (e.g. gender, age, mental health condition), setting (e.g. community, inpatient), indicator (e.g. low mood, aggression), primary outcome (e.g. death, suicide, worsening of symptoms) and main results will be extracted from each study. Two reviewers will independently extract the information. Any potential disagreements will be resolved through group discussion until an overall consensus is obtained; a third reviewer (PA) will be consulted if necessary.

### Data appraisal

Two independent reviewers will appraise studies against the Newcastle-Ottawa Scale (NOS), the recommended tool for assessing quality in non-randomised studies (i.e. cohort and case-control studies) [24, 25]. An amended version of the NOS will be used to assess quality in cross-sectional studies [26]. Each study will be assessed against criteria applicable to each study design, but all will broadly cover the selection of study groups, comparability of these groups and whether the anticipated outcome has been ascertained. Studies will not be excluded based on quality in order to provide a thorough overview of the area.

### Data synthesis

A narrative synthesis approach will be used to analyse the data due to the probable heterogeneous nature of the retrieved studies; therefore, a meta-analysis is unlikely. However, in attempt to assess this possibility, heterogeneity will be assessed using the Q statistic based on the *χ*^2^ test. If homogeneity of the outcomes are sufficient to conduct this analysis, then a random effects model will be used. The main output from this study is likely to be descriptive and will be displayed using an evidence map.

### Write up, dissemination and use of review

The systematic review will be written up according to the PRISMA guidelines to ensure systematic review rigour and be disseminated to local young adult advisory groups (e.g. McPin Foundation). Young adults within these groups will help disseminate to other patient and public groups through discussion at events. The findings will be used to help support the development of future work helping to detect mental and physical health deterioration in young patients.

### Patient and public involvement

**Fig. 1:**
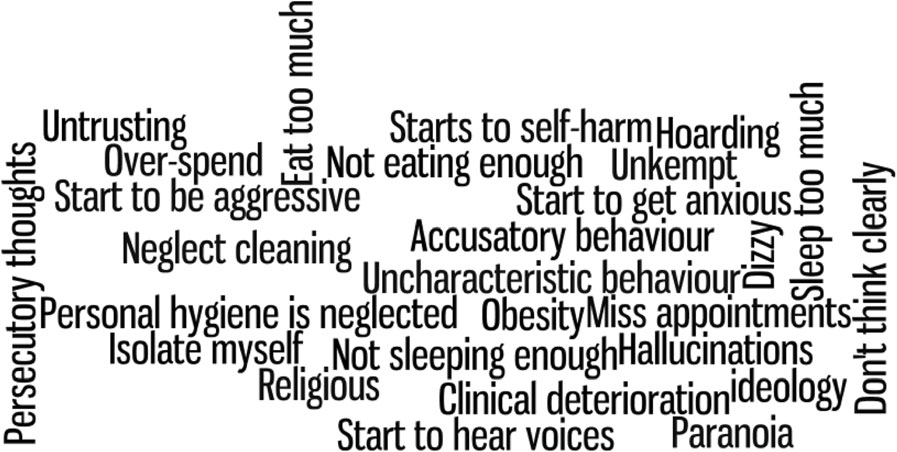
Service users account of “signs of deterioration”

This systematic review is funded by a programme grant from The National Institute for Health Research (NIHR). The INVOLVE advisory group, also funded by NIHR, centres on building patient and public involvement (PPI) into all research projects including systematic reviews [27]. Whilst some find incorporating PPI in systematic reviews challenging, there is evidence to suggest involvement is effective in helping to refine the research question, search terms and selection criteria [28]. Service users have already been approached to help guide the topic area, research question, search terms and inclusion and exclusion criteria. Three service users responded and a 2-h working group was set up. The researcher introduced the topic, proposed research area and asked them to consider answers to three main questions: (1) what does deterioration mean to you? (2) How would you know if someone’s mental health is deteriorating? (3) What can be done to help detect deterioration? The original research question was: “What are the indicators of deterioration in mental healthcare?” Service users were asked to give their initial thoughts, if it could be changed and, if so, what changed could be made. Service users subsequently changed the research question to reflect both patients and individuals who were unknown to mental health services (e.g. young adults and students) and simpler terminology. The interim research question was: “What are the signs of deterioration in people with mental health problems?” This question was further changed to reflect the existing research gap and finalised to be: “What are the signs of deterioration in young adults with serious mental health problems?” This discussion also resulted in a list of possible search terms associated with signs of deterioration (Fig. 1). Whilst informative, the initial scoping review of these terms combined with other terms identified from the literature made the scoping exercise complex; therefore, whereas the research question was changed, the search terms were not incorporated into the final search strategy. The removal of these terms from the final search strategy was also agreed by PA, EC, LH and two resident librarians.

## Discussion

Keeping young patients safe from harm is a key healthcare priority. Identifying indicators of deterioration in patients with SMI is therefore paramount. Subsequent identification may inform the adoption and/or design of an intervention that would enable young adults to self-manage their own mental health; they could know when their symptoms deteriorate and when help is required. Indeed, healthcare professionals or family members or friends of the patients could also be more informed to intervene early.

This protocol describes a systematic review of observational studies that examine the signs for mental and physical deterioration in young adults with SMI. To our knowledge, no other systematic review has examined this area within one review. This systematic review will provide an overview of the aims, methods and results; critical appraisal; and a future research agenda. However, as this review is intentionally exploratory in nature, there are likely to be some difficulties. Firstly, it is likely that studies will be heterogeneous in design and therefore we anticipate assessing study quality will be problematic. As such, it may be more appropriate to use a critical appraisal tool that can assess quality across different research designs. For example, Hawker’s [29] tool accounts for quality regardless of research design.

Secondly, finalising selection criteria will also be difficult because of the varying population, definitions of serious mental illness and types of mental and physical health deterioration to be included in the review. Moreover, the complexity of including mental and physical deterioration in a population who may or may not have been diagnosed with a serious mental health condition may further complicate the search strategy.

## Additional files

Additional file 1: PRISMA-P (Preferred Reporting Items for Systematic review and Meta-Analysis Protocols) 2015 checklist. (DOC 83 kb)

Additional file 2: Full search strategy. (DOCX 45 kb)

## Abbreviations

CINAHL: Cumulative Index to Nursing and Allied Health Literature
CVD: Cardiovascular disease
GP: General practitioner
HMIC: Health Management Information Consortium
NIHR: National Institute for Health Research
NOS: Newcastle-Ottawa Scale
PPI: Patient and public involvement
PRISMA: Preferred reporting items for systematic reviews and meta-analyses
SMI: Serious mental illness
SPSS: Statistical Package for Social Sciences
