# Supplementary material for: Indicators of deterioration in young adults with serious mental illness: a systematic review protocol
Source: Syst Rev. 2018 Aug 16;7:123. doi: 10.1186/s13643-018-0781-y (PMC6097392; doi:10.1186/s13643-018-0781-y)
Supplement: Supplementary file 2 — Full search strategy. (DOCX 45 kb) [file 13643_2018_781_MOESM2_ESM.docx]

| **Additional file 2: Full search strategy** | |
| --- | --- |
|  | |
| Medline | |
| **#** | **Terms** |
| 1 | Exp young adult/ |
| 2 | Exp adolescent/ |
| 3 | young adult*.ti,ab,kw. |
| 4 | adolescen*.ti,ab,kw. |
| 5 | young person*.ti,ab,kw. |
| 6 | young people.ti,ab,kw. |
| 7 | youth*.ti,ab,kw |
| 8 | Exp transtition to adult care/ |
| 9 | ((transition* or transfer* or hand over or handover or handoff) adj5 (adult care or adult services)).ti,ab,kw. |
| **10** | **1 OR 2 OR 3 OR 4 OR 5 OR 6 OR 7 OR 8 OR 9** |
| 11 | Exp bipolar disorder/ |
| 12 | bipolar disorder*.ti,ab,kw. |
| 13 | Exp schizophrenia/ |
| 14 | schizophrenia.ti,ab,kw. |
| 15 | Exp psychotic disorder/ |
| 16 | psychotic disorder*.ti,ab,kw. |
| 17 | Exp major depressive disorder/ |
| 18 | major depression.ti,ab,kw. |
| 19 | major depressive disorder*.ti,ab,kw |
| 20 | exp depressive disorder/ |
| 21 | exp depression/ |
| 22 | depression.ti,ab,kw. |
| 23 | schizoaffective.ti,ab,kw. |
| 24 | psychosis.ti,ab,kw. |
| 25 | mania.ti,ab,kw. |
| 26 | serious mental illness*.ti,ab.tw. |
| **27** | **11 OR 12 OR 13 OR 14 OR 15 OR 16 OR 17 OR 18 OR 19 OR 20 OR 21 OR 22 OR 23 OR 24 OR 25 OR 26** |
| 28 | indicat*.ti,ab.kw |
| 29 | warn*.ti,ab,kw. |
| 30 | MEWS.ti,ab,kw. |
| 31 | predict*.ti,ab.kw. |
| 32 | detect*.ti,ab,kw. |
| 33 | sign*.ti,ab,kw. |
| 34 | measure*.ti,ab,kw. |
| 35 | gauge.ti,ab,kw. |
| 36 | index.ti,ab,kw. |
| 37 | criteria.ti,ab,kw. |
| 38 | highlight.ti,ab,kw. |
| 39 | monitor*.ti,ab,kw. |
| 40 | symptom*.ti,ab,kw. |
| 41 | signal.ti,ab,kw. |
| 42 | diagnos*.ti,ab,kw. |
| 43 | characteristic*.ti,ab,kw. |
| 44 | alarm*.ti,ab,kw. |
| 45 | alert*.ti,ab,kw. |
| 46 | caution.ti,ab,kw. |
| 47 | forewarn*.ti,ab,kw. |
| 48 | trigger*.ti,ab,kw. |
| 49 | risk.ti,ab,kw. |
| 50 | factor*.ti,ab,kw. |
| **51** | **28 OR 29 OR 30 OR 31 OR 32 OR 33 OR 34 OR 35 OR 36 OR 37 OR 38 OR 39 OR 40 OR 41 OR 42 OR 43 OR 44 OR 45 OR 46 OR 47 OR 49 OR 50** |
| 52 | declin*.ti,ab,kw. |
| 53 | deteriorat*ti,ab,kw. |
| 54 | worse*.ti,ab,kw. |
| 55 | downfall.ti,ab,kw. |
| 56 | weak*.ti,ab,kw. |
| 57 | descen*.ti,ab,kw. |
| 58 | laps*.t,ab,kw. |
| 59 | dip*. |
| **60** | **52 OR 53 OR 54 OR 55 OR 56 OR 57 OR 58 OR 59** |
| 61 | exp patient/ |
| 62 | patient*.ti,ab,kw. |
| 63 | inpatient*.ti,ab,kw. |
| 64 | in-patient*.ti,ab,kw. |
| 65 | outpatient*.ti,ab,kw. |
| 66 | out-patient*.ti,ab,kw. |
| **67** | **61 or 62 or 63 or 64 or 65 or 66** |
| **68** | **10 AND 27 AND 51 AND 60 AND 67** |
| **69** | **LIMIT 68 t yr="1991-Current"** |

Embase

| **#** | **Terms** |
| --- | --- |
| 1 | Exp young adult/ |
| 2 | Exp adolescent/ |
| 3 | young adult*.ti,ab,kw. |
| 4 | adolescen*.ti,ab,kw. |
| 5 | young person*.ti,ab,kw. |
| 6 | young people.ti,ab,kw. |
| 7 | youth*.ti,ab,kw |
| 8 | Exp transition to adult care/ |
| 9 | ((transition* or transfer* or hand over or handover or handoff) adj5 (adult care or adult services)).ti,ab,kw. |
| **10** | **1 OR 2 OR 3 OR 4 OR 5 OR 6 OR 7 OR 8 OR 9** |
| 11 | Exp bipolar disorder/ |
| 12 | bipolar disorder*.ti,ab,kw. |
| 13 | Exp schizophrenia/ |
| 14 | schizophrenia.ti,ab,kw. |
| 15 | Exp psychosis/(psychotic disorders on search) |
| 16 | psychotic disorder*.ti,ab,kw. |
| 17 | Exp major depression/(major depressive disorder) |
| 18 | major depressive disorder*.ti,ab,kw |
| 19 | exp depressive disorder/ |
| 20 | exp depression/ |
| 21 | major depression.ti,ab,kw. |
| 22 | depression.ti,ab,kw. |
| 23 | schizoaffective.ti,ab,kw. |
| 24 | psychosis.ti,ab,kw. |
| 25 | mania.ti,ab,kw. |
| 26 | serious mental illness*.ti,ab.tw. |
| **27** | **11 OR 12 OR 13 OR 14 OR 15 OR 16 OR 17 OR 18 OR 19 OR 20 OR 21 OR 22 OR 23 OR 24 OR 25 OR 26** |
| 28 | indicat*.ti,ab.kw |
| 29 | warn*.ti,ab,kw. |
| 30 | MEWS.ti,ab,kw. |
| 31 | predict.ti,ab.kw. |
| 32 | detect*.ti,ab,kw. |
| 33 | sign*.ti,ab,kw. |
| 34 | measure*.ti,ab,kw. |
| 35 | gauge.ti,ab,kw. |
| 36 | index.ti,ab,kw. |
| 37 | criteria.ti,ab,kw. |
| 38 | highlight.ti,ab,kw. |
| 39 | monitor.ti,ab,kw. |
| 40 | symptom*.ti,ab,kw. |
| 41 | signal.ti,ab,kw. |
| 42 | diagnos*.ti,ab,kw. |
| 43 | characteristic*.ti,ab,kw. |
| 44 | alarm.ti,ab,kw. |
| 45 | alert*.ti,ab,kw. |
| 46 | caution.ti,ab,kw. |
| 47 | forewarn*.ti,ab,kw. |
| 48 | trigger*.ti,ab,kw. |
| 49 | risk.ti,ab,kw. |
| 50 | factor*.ti,ab,kw. |
| **51** | **28 OR 29 OR 30 OR 31 OR 32 OR 33 OR 34 OR 35 OR 36 OR 37 OR 38 OR 39 OR 40 OR 41 OR 42 OR 43 OR 44 OR 45 OR 46 OR 47 OR 49 OR 50** |
| 52 | declin*.ti,ab,kw. |
| 53 | deteriorat*ti,ab,kw. |
| 54 | worse*.ti,ab,kw. |
| 55 | downfall.ti,ab,kw. |
| 56 | weak*.ti,ab,kw. |
| 57 | descen*.ti,ab,kw. |
| 58 | laps*.t,ab,kw. |
| 59 | dip*. |
| **60** | **52 OR 53 OR 54 OR 55 OR 56 OR 57 OR 58 OR 59** |
| 61 | exp patient/ |
| 62 | patient*.ti,ab,kw. |
| 63 | inpatient*.ti,ab,kw. |
| 64 | in-patient*.ti,ab,kw. |
| 65 | outpatient*.ti,ab,kw. |
| 66 | out-patient*.ti,ab,kw. |
| **67** | **61 OR 62 OR 63 OR 64 OR 65 OR 66** |
| **68** | **10 AND 27 AND 51 AND 60 AND 67** |
| **69** | **LIMIT 68 t yr="1991-Current"** |

PsycInfo

| **#** | **Terms** |
| --- | --- |
| 1 | Exp young adult/ |
| 2 | Exp adolescent/ |
| 3 | young adult*.ti,ab,kw. |
| 4 | adolescen*.ti,ab,kw. |
| 5 | young person*.ti,ab,kw. |
| 6 | young people.ti,ab,kw. |
| 7 | youth*.ti,ab,kw |
| 8 | Exp transition to adult care/ |
| 9 | ((transition* or transfer* or hand over or handover or handoff) adj5 (adult care or adult services)).ti,ab,kw. |
| **10** | **1 OR 2 OR 3 OR 4 OR 5 OR 6 OR 7 OR 8 OR 9** |
| 11 | Exp bipolar disorder/ |
| 12 | bipolar disorder*.ti,ab,kw. |
| 13 | Exp schizophrenia/ |
| 14 | schizophrenia.ti,ab,kw. |
| 15 | Exp psychosis/(psychotic disorders on search) |
| 16 | psychotic disorder*.ti,ab,kw. |
| 17 | Exp major depression/(major depressive disorder) |
| 18 | major depressive disorder*.ti,ab,kw |
| 19 | exp depressive disorder/ |
| 20 | exp depression/ |
| 21 | major depression.ti,ab,kw. |
| 22 | depression.ti,ab,kw. |
| 23 | schizoaffective.ti,ab,kw. |
| 24 | psychosis.ti,ab,kw. |
| 25 | mania.ti,ab,kw. |
| 26 | serious mental illness*.ti,ab.tw. |
| **27** | **11 OR 12 OR 13 OR 14 OR 15 OR 16 OR 17 OR 18 OR 19 OR 20 OR 21 OR 22 OR 23 OR 24 OR 25 OR 26** |
| 28 | indicat*.ti,ab.kw |
| 29 | warn*.ti,ab,kw. |
| 30 | MEWS.ti,ab,kw. |
| 31 | predict.ti,ab.kw. |
| 32 | detect*.ti,ab,kw. |
| 33 | sign*.ti,ab,kw. |
| 34 | measure*.ti,ab,kw. |
| 35 | gauge.ti,ab,kw. |
| 36 | index.ti,ab,kw. |
| 37 | criteria.ti,ab,kw. |
| 38 | highlight.ti,ab,kw. |
| 39 | monitor.ti,ab,kw. |
| 40 | symptom*.ti,ab,kw. |
| 41 | signal.ti,ab,kw. |
| 42 | diagnos*.ti,ab,kw. |
| 43 | characteristic*.ti,ab,kw. |
| 44 | alarm.ti,ab,kw. |
| 45 | alert*.ti,ab,kw. |
| 46 | caution.ti,ab,kw. |
| 47 | forewarn*.ti,ab,kw. |
| 48 | trigger*.ti,ab,kw. |
| 49 | risk.ti,ab,kw. |
| 50 | factor*.ti,ab,kw. |
| **51** | **28 OR 29 OR 30 OR 31 OR 32 OR 33 OR 34 OR 35 OR 36 OR 37 OR 38 OR 39 OR 40 OR 41 OR 42 OR 43 OR 44 OR 45 OR 46 OR 47 OR 48 OR 49 OR 50** |
| 52 | declin*.ti,ab,kw. |
| 53 | deteriorat*ti,ab,kw. |
| 54 | worse*.ti,ab,kw. |
| 55 | downfall.ti,ab,kw. |
| 56 | weak*.ti,ab,kw. |
| 57 | descen*.ti,ab,kw. |
| 58 | laps*.ti,ab,kw. |
| 59 | dip*.ti,ab,kw. |
| **60** | **52 OR 53 OR 54 OR 55 OR 56 OR 57 OR 58 OR 59** |
| 61 | Exp patients/ |
| 62 | Patient*.ti,ab,kw. |
| 63 | In-patient*ti,ab,kw. |
| 64 | Inpatient*.ti,ab,kw. |
| 65 | Out-patient*.ti,ab,kw. |
| 66 | Outpatient*.ti,ab,kw. |
| **67** | **61 OR 62 OR 63 OR 64 Or 65 OR 66** |
| **68** | **10 AND 27 AND 51 AND 60 AND 67** |
| **69** | **LIMIT 68 t yr="1991-Current"** |

HMIC

| **#** | **Terms** |
| --- | --- |
| 1 | Exp young adult/ |
| 2 | Exp adolescent/ |
| 3 | young adult*.ti,ab,kw. |
| 4 | adolescen*.ti,ab,kw. |
| 5 | young person*.ti,ab,kw. |
| 6 | young people.ti,ab,kw. |
| 7 | youth*.ti,ab,kw |
| 8 | Transition to adult care/ |
| 9 | ((transition* or transfer* or hand over or handover or handoff) adj5 (adult care or adult services)).ti,ab,kw. |
| **10** | **1 OR 2 OR 3 OR 4 OR 5 OR 6 OR 7 OR 8 OR 9** |
| 11 | exp Bipolar Disorder/ |
| 12 | bipolar disorder*.ti,ab,kw. |
| 13 | exp Schizophrenia/ |
| 14 | schizophrenia.ti,ab,kw. |
| 15 | exp Psychotic Disorders/ |
| 16 | exp psychosis/ |
| 17 | psychotic disorder*.ti,ab,kw. |
| 18 | exp Depressive Disorder, Major/ |
| 19 | exp major depression/ |
| 20 | major depression.ti,ab,kw. |
| 21 | major depressive disorder*.ti,ab,kw. |
| 22 | exp depressive disorder/ |
| 23 | exp depression/ |
| 24 | depression.ti,ab,kw. |
| 25 | schizoaffective.ti,ab,kw. |
| 26 | psychosis.ti,ab,kw. |
| 27 | mania.ti,ab,kw. |
| 28 | Serious mental illness*.ti,ab,kw. |
| **29** | **11 OR 12 OR 13 OR 14 OR 15 OR 16 OR 17 OR 18 OR 19 OR 20 OR 21 OR 22 OR 23 OR 24 OR 25 Or 26 OR 27 OR 28** |
| 30 | indicat*.ti,ab.kw |
| 31 | warn*.ti,ab,kw. |
| 32 | MEWS.ti,ab,kw. |
| 33 | predict.ti,ab.kw. |
| 34 | detect*.ti,ab,kw. |
| 35 | sign*.ti,ab,kw. |
| 36 | measure*.ti,ab,kw. |
| 37 | gauge.ti,ab,kw. |
| 38 | index.ti,ab,kw. |
| 39 | criteria.ti,ab,kw. |
| 40 | highlight*.ti,ab,kw. |
| 41 | monitor*.ti,ab,kw. |
| 42 | symptom*.ti,ab,kw. |
| 43 | signal.ti,ab,kw. |
| 44 | diagnos*.ti,ab,kw. |
| 45 | characteristic*.ti,ab,kw. |
| 46 | alarm*.ti,ab,kw. |
| 47 | alert*.ti,ab,kw. |
| 48 | caution.ti,ab,kw. |
| 49 | forewarn*.ti,ab,kw. |
| 50 | trigger*.ti,ab,kw. |
| 51 | risk.ti,ab,kw. |
| 52 | factor*.ti,ab,kw. |
| **53** | **30 OR 31 OR 32 OR 33 OR 34 OR 35 OR 36 OR 37 OR 38 OR 39 OR 40 OR 41 OR 42 OR 43 OR 44 OR 45 OR 46 OR 47 OR 48 OR 49 OR 50 OR 51 OR 52** |
| 54 | declin*.ti,ab,kw. |
| 55 | deteriorat*ti,ab,kw. |
| 56 | worse*.ti,ab,kw. |
| 57 | downfall.ti,ab,kw. |
| 58 | weak*.ti,ab,kw. |
| 59 | descen*.ti,ab,kw. |
| 60 | laps*.ti,ab,kw. |
| 61 | dip*.ti,ab,kw. |
| **62** | **54 OR 55 OR 57 OR 58 OR 59 OR 60 OR 61** |
| 63 | exp patients/ |
| 64 | patient*.ti,ab,kw. |
| 65 | inpatient*.ti,ab,kw. |
| 66 | in-patient*.ti,ab,kw. |
| 67 | outpatient*.ti,ab,kw. |
| 68 | out-patient*.ti,ab,kw. |
| **69** | **50 OR 51 OR 52 OR 53 OR 54 OR 55 OR 56 OR 57** |
| **70** | **10 AND 25 AND 49 AND 58** |
| **71** | **LIMIT 70 t yr="1991-Current"** |

Web of science

| **#** | **Terms** |
| --- | --- |
| 1 | TS=young adult* |
| 2 | TS=adolescen* |
| 3 | TS=young person* |
| 4 | TS=young people |
| 5 | TS=youth* |
| 6 | TS=(transition* or transfer* or "hand over" or handover or handoff "NEAR/4" ("adult care" or "adult services")) |
| **7** | **#1 OR #2 OR #3 OR #4 OR #5 OR #6** |
| 8 | TS=bipolar disorder* |
| 9 | TS=schizophrenia |
| 10 | TS=psychotic disorder* |
| 11 | TS=major depressive disorder* |
| 12 | TS=major depression |
| 13 | TS=depression |
| 14 | TS=schizoaffective |
| 15 | TS=psychosis |
| 16 | TS=mania |
| 17 | TS=serious mental illness* |
| **18** | **#8 OR #9 OR #10 OR #11 OR #12 OR #13 OR #14 OR #15 OR #16 OR #17** |
| 19 | indicat* |
| 20 | TS=warn* |
| 21 | TS=MEWS |
| 22 | TS=predict |
| 23 | TS=detect* |
| 24 | TS=sign* |
| 25 | TS=measure* |
| 26 | TS=gauge |
| 27 | TS=index |
| 28 | TS=criteria |
| 29 | TS=highlight* |
| 30 | TS=monitor* |
| 31 | TS=symptom* |
| 32 | TS=signal |
| 33 | TS=diagnos* |
| 34 | TS=characteristic* |
| 35 | TS=alarm |
| 36 | TS=alert* |
| 37 | TS=caution |
| 38 | TS=forewarn* |
| 39 | TS=trigger* |
| 40 | TS=risk |
| 41 | TS=factor* |
| **42** | **#19 OR #20 OR #21 OR #22 OR #23 OR #24 OR #25 OR #26 OR #27 OR #28 OR #29 OR #30 OR #31 OR #32 OR #33 OR #34 OR #35 OR #36 OR #37 OR #38 OR #39 OR #40 OR #41** |
| 43 | TS=declin* |
| 44 | TS=deteriorat* |
| 45 | TS=worse* |
| 46 | TS=downfall |
| 47 | TS=weak* |
| 48 | TS=descen* |
| 49 | TS=laps* |
| 50 | TS=dip* |
| **51** | **#43 OR #44 OR #45 OR #46 OR #47 OR #48 OR #49 OR #50** |
| 52 | TS=patient* |
| 53 | TS=inpatient* |
| 54 | TS=in-patient* |
| 55 | TS=outpatient* |
| 56 | TS=out-patient* |
| 57 | TS=patient* |
| **58** | **#52 OR #53 OR #54 or #55 OR #56 or #57** |
| **59** | **7 AND 18 AND 42 AND 51 AND 58** |
| **60** | **LIMIT 59 t yr="1991-Current"** |

CINHAL

| **#** | **Terms** |
| --- | --- |
| 1 | MH young adult |
| 2 | MH adolescent+ |
| 3 | TI young adult* OR AB young adult* OR MW young adult* |
| 4 | TI adolescen* OR AB adolescen* OR MW adolescen* |
| 5 | TI young person* OR AB young person* OR MW young person* |
| 6 | TI young people OR AB young people OR MW young people |
| 7 | MH Youth |
| 8 | MH transition to adult care/ |
| 9 | ((transition* or transfer* or hand over or handover or handoff) adj5 (adult care or adult services)).ti,ab,kw. |
| **10** | **1 OR 2 OR 3 OR 4 OR 5 OR 6 OR 7 OR 8 OR 9** |
| 11 | MH bipolar disorder+ |
| 12 | TI bipolar disorder* OR AB bipolar disorder* MW bipolar disorder* |
| 13 | MH schizophrenia+ |
| 14 | TI schizophrenia AB schizophrenia MW schizophrenia |
| 15 | MH psychotic disorders+ |
| 16 | TI psychotic disorder* OR AB psychotic disorder* OR MW psychotic disorder* |
| 17 | MH major depression+ |
| 18 | TI major depressive disorder* AB major depressive disorder* OR MW major depressive disorder* |
| 19 | TI major depression AB major depression MW major depression |
| 20 | TI depression AB depression MW depression |
| 21 | MH depression+ |
| 22 | TI schizoaffective AB schizoaffective MW schizoaffective |
| 23 | TI psychosis AB psychosis MW psychosis |
| 24 | TI mania AB mania MW mania |
| 25 | TI serious mental illness AB serious mental illness MW serious mental illness |
| **26** | **11 OR 12 OR 13 OR 14 OR 15 OR 16 OR 17 OR 18 OR 19 OR 20 OR 21 OR 22 OR 23 OR 24 OR 25** |
| 27 | TI indicat* AB indicat* MW indicat* |
| 28 | TI warn* AB indicat* MW indicat* |
| 29 | TI MEWS AB MEWS MW MEWS |
| 30 | TI predict* AB predict* MW predict* |
| 31 | TI detect* AB detect* MW detect* |
| 32 | TI sign* AB sign* MW sign* |
| 33 | TI measure* AB measure* MW measure* |
| 34 | TI gauge AB gauge MW gauge |
| 35 | TI index AB index MW index |
| 36 | TI criteria AB criteria MW criteria |
| 37 | TI highlight AB highlight MW highlight |
| 38 | TI monitor AB monitor MW monitor |
| 39 | TI symptom* AB symptom* MW symptom* |
| 40 | TI signal* AB signal* MW signal* |
| 41 | TI diagnos* AB diagnos* MW diagnos* |
| 42 | TI characteristic* AB characteristic* MW characteristic* |
| 43 | TI alarm AB alarm MW alarm |
| 44 | TI alert* AB alert MW alert |
| 45 | TI caution AB caution MW caution |
| 46 | TI forewarn* AB forewarn* MW forewarn* |
| 47 | TI trigger* AB trigger* MW trigger* |
| 48 | TI risk AB risk MW risk |
| 49 | TI factor* AB factor* MW factor* |
| **50** | **26 OR 27 OR 28 OR 29 OR 30 OR 31 OR 32 OR 33 OR 34 OR 35 OR 36 OR 37 OR 38 OR 39 OR 40 OR 41 OR 42 OR 43 OR 44 OR 45 OR 46 OR 47 OR 48 OR 49** |
| 51 | TI declin* AB declin* MW declin* |
| 52 | TI deteriorat* AB deteriorat* MW deteriorat* |
| 53 | TI worse* AB worse* MW worse* |
| 54 | TI downfall AB downfall MW downfall |
| 55 | TI weak* AB weak* MW weak* |
| 56 | TI descen* AB descen* MW descen* |
| 57 | TI laps* AB laps* MW laps* |
| 58 | TI dip* AB dip* MW* dip* |
| **59** | **51 OR 52 OR 53 OR 54 OR 55 OR 56 OR 57 OR 58** |
| 60 | MH patients+ |
| 61 | TI patient* AB patient* MW patient* |
| 62 | TI inpatient* AB inpatient* MW inpatient* |
| 63 | TI in-patient* AB in-patient* MW in-patient* |
| 64 | TI outpatient* AB outpatient* MW outpatient* |
| 65 | TI out-patient* AB out-patient* MW out-patient* |
| **66** | **60 OR 61 OR 62 OR 63 OR 64 OR 65** |
| **67** | **10 AND 26 AND 50 AND 59 AND 65** |
| **68** | **LIMIT 67 t yr="1991-Current"** |

The Cochrane Library

| **#** | **Terms** |
| --- | --- |
| 1 | Exp young adult/ |
| 2 | Exp adolescent/ |
| 3 | young adult*.ti,ab,kw. |
| 4 | adolescen*.ti,ab,kw. |
| 5 | young person*.ti,ab,kw. |
| 6 | young people.ti,ab,kw. |
| 7 | youth*.ti,ab,kw |
| 8 | Exp transtition to adult care/ |
| 9 | ((transition* or transfer* or hand over or handover or handoff) adj5 (adult care or adult services)).ti,ab,kw. |
| **10** | **#1 OR #2 OR #3 OR #4 OR #5 OR #6 OR #7 OR #8 OR #9** |
| 11 | Exp bipolar disorder/ |
| 12 | bipolar disorder*.ti,ab,kw. |
| 13 | Exp schizophrenia/ |
| 14 | schizophrenia.ti,ab,kw. |
| 15 | Exp psychotic disorder/ |
| 16 | psychotic disorder*.ti,ab,kw. |
| 17 | Exp major depressive disorder/ |
| 18 | major depression.ti,ab,kw. |
| 19 | major depressive disorder*.ti,ab,kw |
| 20 | exp depressive disorder/ |
| 21 | exp depression/ |
| 22 | depression.ti,ab,kw. |
| 23 | schizoaffective.ti,ab,kw. |
| 24 | psychosis.ti,ab,kw. |
| 25 | mania.ti,ab,kw. |
| 26 | serious mental illness*.ti,ab.tw. |
| **27** | **#11 OR #12 OR #13 OR #14 OR #15 OR #16 OR #17 OR #18 OR #19 OR #20 OR #21 OR #22 OR #23 OR #24 OR #25 OR #26** |
| 28 | indicat*.ti,ab.kw |
| 29 | warn*.ti,ab,kw. |
| 30 | MEWS.ti,ab,kw. |
| 31 | predict*.ti,ab.kw. |
| 32 | detect*.ti,ab,kw. |
| 33 | sign*.ti,ab,kw. |
| 34 | measure*.ti,ab,kw. |
| 35 | gauge.ti,ab,kw. |
| 36 | index.ti,ab,kw. |
| 37 | criteria.ti,ab,kw. |
| 38 | highlight.ti,ab,kw. |
| 39 | monitor*.ti,ab,kw. |
| 40 | symptom*.ti,ab,kw. |
| 41 | signal.ti,ab,kw. |
| 42 | diagnos*.ti,ab,kw. |
| 43 | characteristic*.ti,ab,kw. |
| 44 | alarm*.ti,ab,kw. |
| 45 | alert*.ti,ab,kw. |
| 46 | caution.ti,ab,kw. |
| 47 | forewarn*.ti,ab,kw. |
| 48 | trigger*.ti,ab,kw. |
| 49 | risk.ti,ab,kw. |
| 50 | factor*.ti,ab,kw. |
| 51 | **#28 OR #29 OR #30 OR #31 OR #32 OR #33 OR #34 OR #35 OR #36 OR #37 OR #38 OR #39 OR #40 OR #41 OR #42 OR #43 OR #44 OR #45 OR #46 OR #47 OR #48 OR #49 OR #50** |
| 52 | declin*.ti,ab,kw. |
| 53 | deteriorat*ti,ab,kw. |
| 54 | worse*.ti,ab,kw. |
| 55 | downfall.ti,ab,kw. |
| 56 | weak*.ti,ab,kw. |
| 57 | descen*.ti,ab,kw. |
| 58 | laps*.t,ab,kw. |
| 59 | dip*.ti, ab, kw |
| **60** | **#52 OR #53 OR #54 OR #55 OR #56 OR #57 OR #58 OR #59** |
| 61 | exp patients/ |
| 62 | patient*.ti,ab,kw. |
| 63 | inpatient*.ti,ab,kw. |
| 64 | in-patient*.ti,ab,kw. |
| 65 | outpatient*.ti,ab,kw. |
| 66 | out-patient*.ti,ab,kw. |
| **67** | **#61 or #62 or #63 or #64 or #65 or #66** |
| **68** | **#10 and #27 and #51 and #60 and #67** |
| **69** | **LIMIT 67 t yr="1991-Current"** |
